# Effects and Mechanisms of *Rhus chinensis* Mill. Fruits on Suppressing RANKL-Induced Osteoclastogenesis by Network Pharmacology and Validation in RAW264.7 Cells

**DOI:** 10.3390/nu14051020

**Published:** 2022-02-28

**Authors:** Yue Zheng, Lei Zhao, Junjie Yi, Shengbao Cai

**Affiliations:** 1 Faculty of Food Science and Engineering, Kunming University of Science and Technology, Kunming 650500, China; zheng_yue21@163.com (Y.Z.); junjieyi@kust.edu.cn (J.Y.); 2 Beijing Engineering and Technology Research Center of Food Additives, Beijing Technology and Business University, Beijing 100048, China; zhaolei@th.btbu.edu.cn

**Keywords:** Chinese sumac, osteoclast, osteoporosis, network pharmacology, phenolic compounds

## Abstract

*Rhus chinensis* Mill. fruits are a kind of widely distributed edible seasoning, which have been documented to possess a variety of biological activities. However, its inhibitory effect on osteoclast formation has not been determined. The objective of this study was to evaluate the effect of the fruits on osteoclast differentiation of RAW264.7 cells, induced by receptor activator of nuclear factor-κB ligand (RANKL) and to illuminate the potential mechanisms using network pharmacology and western blots. Results showed that the extract containing two organic acids and twelve phenolic substances could effectively inhibit osteoclast differentiation in RANKL-induced RAW264.7 cells. Network pharmacology examination and western blot investigation showed that the concentrate essentially decreased the expression levels of osteoclast-specific proteins, chiefly through nuclear factor kappa-B, protein kinase B, and mitogen-activated protein kinase signaling pathways, particularly protein kinase B α and mitogen-activated protein kinase 1 targets. Moreover, the extract likewise directly down regulated the expression of cellular oncogene Fos and nuclear factor of activated T-cells cytoplasmic 1 proteins. Citric acid, quercetin, myricetin-3-*O*-galactoside, and quercetin-3-*O*-rhamnoside were considered as the predominant bioactive ingredients. Results of this work may provide a scientific basis for the development and utilization of *R. chinensis* fruits as a natural edible material to prevent and/or alleviate osteoporosis-related diseases.

## 1. Introduction

Human bones are constantly modified, and the dynamic balance of osteoblasts and osteoclasts is an important factor to maintain human bone health [1]. However, when this homeostasis becomes imbalanced, it can induce the growth of bone-solubilizing diseases, such as osteoporosis (OP) [2,3], osteosclerosis [4], etc. The main manifestations of OP are exacerbation of bone organization microarchitecture, reduction of bone density and an increase in susceptibility to fragile fractures [5]. OP can be mainly divided into primary OP and secondary OP, and the underlying pathogenesis is that bone loss is faster than bone formation [6,7,8]. With the development of medical technology and the standard of living, the average life expectancy of people is increasing which is accelerating the aging of society, resulting in a higher prevalence of OP [9]. The excessive activation of osteoclasts assumes a critical part in the pathology of OP [10]. Therefore, the inhibition of osteoclast differentiation has been considered as a potential therapy for the treatment and/or prevention of OP with few side effects.

Osteoclasts are a kind of novel multinucleated cell with bone resorbing capacity that originates from the bone marrow monocyte-macrophage lineage [11]. A major cascade of signals regulating osteoclast maturation and activation is the receptor activator of nuclear factor-κB (RANK)/receptor activator of nuclear factor-κB ligand (RANKL) pathway. RANKL facilitates osteoclast activation by combination with RANK on the cell membrane of osteoclast lineage cells [12]. After activation, the expression of many key transcription activators and enzymes increases, which in turn promote transdifferentiation, multiplication, and multi-nucleation of the osteoclast [4]. RANKL recruits tumor necrosis factor receptor-associated factor 6 (TRAF6) to activate a series of downstream cascades, including NF-κB, Akt, and MAPKs signaling pathways, and then further activates nuclear factor of activated T-cells cytoplasmic 1 (NFATc1) and cellular oncogene Fos (c-Fos), which are the final step for osteoclast formation [13]. Currently, the representative drugs for OP treatment are bisphosphonates and estrogens [14], but these substances inevitably cause varying degrees of side effects and complications in humans, such as osteonecrosis of the jaw and unrepresentative femur fractures [15]. Therefore, it is essential to develop some alternative dietary therapy with fewer side effects to prevent and/or improve OP.

**Figure 1:**
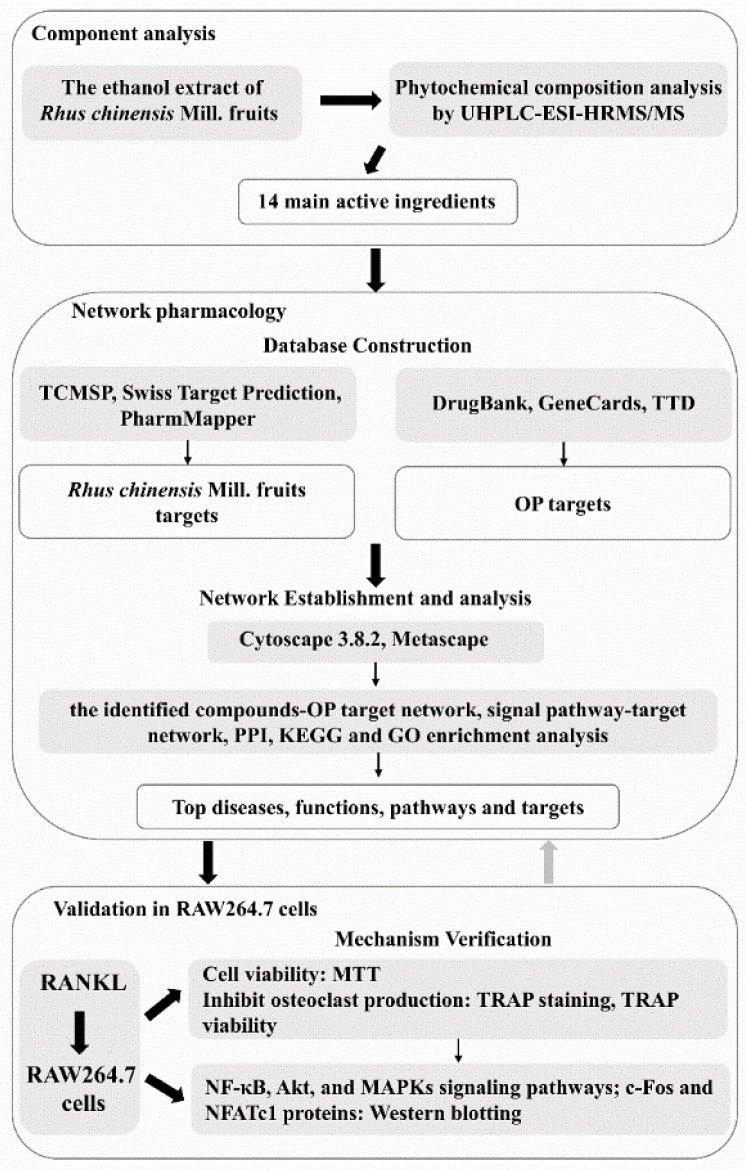
Workflow of the research. OP, osteoporosis; MTT, 3(4,5-Dimethylthiazol-2-yl)-2,5-diphenyltetrazolium bromide; TRAP, tartrate-resistant acid phosphatase; RANKL, nuclear factor-κB ligand; NF-κB, nuclear factor kappa-B; c-Fos, cellular oncogene Fos; NFATc1, nuclear factor of activated T-cells cytoplasmic 1.

Polyphenols are abundant in common edible fruits, vegetables and herbs. They have good antioxidant and anti-inflammatory activities and can maintain the health of the body [16]. At present, many studies have found that plant polyphenols have inhibitory effects on the formation of osteoclasts [17,18]. The study by Thomas et al. [17] showed that TRAP activity, an indicator of osteoclast differentiation, exhibited a downward trend with treatment using tart cherry polyphenols; moreover, result also showed that high doses of tart cherry polyphenols (300 µg/mL) could reduce the production of inflammatory markers, including nitric oxide content, cyclooxygenase 2, in RANKL-induced RAW264.7 cells, thereby reducing the differentiation and resorption activity of osteoclasts. *Aronia melanocarpa* ‘Viking’ (AM) was rich in phenolic compounds, and the study of Ghosh et al. [18], showed that the water and alcohol extracts of AM could inhibit the excessive accumulation of ROS, and the expression of osteoclast-related genes, including integrin β_3_, TRAP, cathepsin K and calcitonin receptor; in addition, both extracts inhibited osteoclastogenesis by acting on the MAPKs pathway and blocking the signaling of c-Fos and NFATc1. *Rhus chinensis* pertains to the genus *Rhus* of the *Anacardiaceae* family, and is extensively distributed in Asia, including China, and Japan [19]. The fruits are commonly used as a kind of appetizer, beverage or natural vinegar by local people [19]. In addition, the fruits are recorded as a traditional herb with a wide range of bioactivities, which can be used to preclude and/or cure some diseases such as jaundice, alcoholism, hepatitis, and inflammatory diseases [20,21]. *Rhus chinensis* Mill. fruits have high nutritional value and are rich in a variety of polyphenols, polyunsaturated fatty acids and other phytochemicals [19]. For example, quercetin, myricetin-3-*O*-galactoside, and quercetin-3-*O*-rhamnoside are polyphenols that are abundant in *R. chinensis* fruits [21,22]. Some studies have reported that quercetin has related activities such as inhibiting osteoclastogenesis [23,24]. Kim et al. [23], found that quercetin could play an immunomodulatory role in interleukin-17 (IL-17) produced osteoclastogenesis. The results of Guo et al. [24], showed that quercetin could inhibit lipopolysaccharide-induced osteoclast bone resorption. Based on the above related findings, we speculated that *R. chinensis* fruits may have a bioactivity that inhibits the differentiation and formation of osteoclasts. However, no study has been carried out to investigate the preventive effects and the underlying mechanisms involved in the differentiation and formation of osteoclasts. The aim of this work was to investigate the preventive effects and potential mechanisms of the ethanolic extract from *R. chinensis* fruits against the differentiation and formation of osteoclasts by using network pharmacology and validation of results using cellular experiments (Figure 1).

## 2. Materials and Methods

### 2.1. Reagents and Chemicals

RANKL was obtained from R&D Systems (Minneapolis, MN, USA). BCA protein assay kit, 3(4,5-Dimethylthiazol-2-yl)-2,5-diphenyltetrazolium bromide (MTT) and tartrate-resistant acid phosphatase (TRAP) activity kits were supplied by Beyotime Biotechnology (Shanghai, China). Penicillin, streptomycin, Dulbecco’s modified Eagle’s medium (DMEM) and fetal bovine serum (FBS) were purchased from Gibco-Invitrogen (Karlsruhe, Baden-Württemberg, Germany). Cell lysis buffer with inhibitors of protease and phosphatase were provided by Nanjing Jiancheng Bioengineering Institute (Nanjing, China). Antibodies used in the current work, including NF-κB, p-IκBα, IκBα, p-JNK (phospho-JNK1-T183/Y185 + JNK2-T183/Y185 + JNK3-T221/Y223), JNK, p-ERK (phospho-ERK1-T202/Y204 + ERK2-T185/Y187), ERK, p38, p-p38 (phospho-p38 MAPK-T180/Y182 Rabbit pAb), Akt, p-Akt (Ser 473) and β-Actin were supplied by Wuhan Abclonal, China, and p-NF-κB, c-Fos, and NFATc1 were obtained from Affinity (Carlsbad, CA, USA).

### 2.2. Sample Preparation

*R. chinensis* fruits were collected by Kunming Plant Classification Biotechnology Co., Ltd. (Kunming, China) from Tengchong County, Yunnan Province, in November 2019. All fruit materials were washed with tap water to remove impurities, dried naturally, and stored at −20 °C. The fruit was then freeze–dried, crushed to pass through a 40 mesh sieve. The 80% ethanolic extract was prepared in accordance with a former report [22] and the extract concentrated by a Heidolph Hei-VAP rotary evaporator (Hei-VAP Advantage, Heidolph, Germany) and freeze-dried.

### 2.3. Characterization of Phytochemical Composition with UHPLC-ESI-HRMS/MS

Phytochemical components in the extract were characterized by using the Ultimate 3000 UHPLC System of Thermo Fisher coupled with a Thermo Fisher Scientific Q-Exactive Orbitrap mass (Bremen, Germany). Phytochemical substances of the ethanolic extract from the fruits were first separated with a Zorbax SB-C18 column (Agilent, 2.1 × 100 mm, 1.7 μm). The injection volume and flow rate were 3 µL and 0.2 mL/min. The column temperature was set at 35 °C. Ultrapure water acidified by 0.1% formic acid (phase A) and acetonitrile (phase B) were applied as mobile phases. The following program was used as gradient elution conditions: 0–2 min, 5% B; 2–10 min, 5–15% B; 10–25 min, 15–30% B; 25–30 min, 30–50% B; 30–32 min, 50–5% B. The mass spectrum conditions were set according to our previous study [25]. The compounds were preliminarily or positively characterized by comparing the mass data of the compounds with the data in the corresponding standards, databases (mass bank) or references. Identified compounds were (semi-) quantified in accordance with the curved line of corresponding standards (or at least similar aglycones).

### 2.4. Network Pharmacology Analysis

For revealing the many potential substances in *R. chinensis* fruits against OP and their mechanism in inhibiting osteoclast differentiation, network pharmacology was applied in the present work. First, the standard structures of the detected components in *R. chinensis* fruits were obtained from PubChem (https://pubchem.ncbi.nlm.nih.gov, accessed on 28 July 2021). Then the Traditional Chinese Medicine Systems Pharmacology Database (TCMSP, http://tcmspw.com/tcmsp.php, accessed on 28 July 2021) [26], Swiss Target Prediction (http://www.swisstargetprediction.cn, accessed on 28 July 2021) [27] and PharmMapper (http://www.lilab-ecust.cn/pharmmapper/, accessed on 28 July 2021) [28] were applied to search and screen the related targets of the bioactive substances. Keyword “osteoporosis”, was used in DrugBank (https://go.drugbank.com, accessed on 28 July 2021) [29], GeneCards (https://genecards.weizmann.ac.il/v3/, accessed on 28 July 2021) [30] and TherapeuticTargetDatabase (TTD, http://db.idrblab.net/ttd/, accessed on 28 July 2021) [31] to gather disease targets. UniProtKB (https://www.uniprot.org/, accessed on 29 July 2021) [32] was applied to obtain the name of the standard target with “Homo sapiens” as the selected organism. The Venn online platform (http://bioinformatics.psb.ugent.be/webtools/Venn/, accessed on 29 July 2021) was applied to intersect the active ingredient targets and disease targets retrieved from the above mentioned database. Then, the common target of active ingredients and diseases were obtained, which was the target of prevention and treatment of osteoporosis by the ethanolic extract of *R. chinensis.* fruits. Cytoscape 3.8.2 software was applied to establish the active ingredient–target network, signal pathway–target network or protein–protein interaction (PPI) network. KEGG and GO enrichment analyses were performed by Metascape (https://metascape.org/gp/index.html#/main/step1, accessed on 30 July 2021). Histograms were plotted by http://www.bioinformatics.com.cn (accessed on 30 July 2021).

### 2.5. Cell Culture and Cytotoxicity Test

RAW264.7 cells obtained from the Kunming cell bank of the Chinese Academy of Sciences (Kunming, China) were incubated in DMEM medium with 10% FBS. The culture temperature was 37 °C and the CO_2_ concentration was 5%. The MTT method was used to evaluate the cytotoxicity of the ethanolic extract at 50 μg/mL or 100 μg/mL with RAW264.7 cells as previously reported [25]. Results showed that both concentrations of the ethanolic extract exhibited no cellular toxicity to RAW264.7 cells.

### 2.6. TRAP Staining

RAW264.7 cells seeded in 96-well plates were set up in accordance with the above concentrations in the following groups: Group K (Control group), Group M (50 ng/mL RANKL), Group RL (50 ng/mL RANKL and 50 µg/mL of extract), and group RH (50 ng/mL RANKL and 100 µg/mL of extract). The solution was changed every other day. After incubation for 5 days, the cells were stained according to the instructions of the TRAP staining kit.

### 2.7. TRAP Viability Examination

RAW264.7 cells seeded into 6-well plates were cultured according to the above method. After being cultured for five days, TRAP activity of each well was detected according to the kit instructions for TRAP viability.

### 2.8. Analysis by Western Blots

RAW264.7 cells seeded into 6-well plates were cultured according to the above method. The solution was replaced every other day for five days. All cells were homogenized in cell lysis buffer with a scientz-IID ultrasonic cell crusher (Ningbo Scientz Biotech Co., Ltd., Ningbo, China). Western blot analyses were carried out according to the previously reported method [19].

### 2.9. Statistical Analysis

All data are expressed as average ± standard deviation (S.D.). The data were evaluated with one-way ANOVA, and the significance (*p* < 0.05) was tested by Tukey’s test. TRAP staining was performed by Media Cybernetics Inc. (Rockville, MD, USA) using the software image-Pro Plus 6.0. Origin 8.5 software was used for data analyses in the current work.

## 3. Results

### 3.1. Phytochemical Composition Analysis

**Figure 2:**
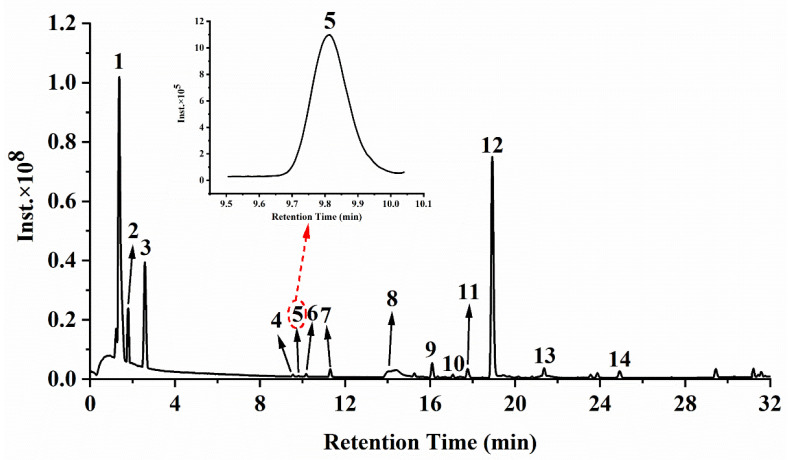
Mass chromatograms of the ethanolic extract from *R. chinensis* fruits in negative mode. Peaks 1–14 refer to the corresponding compounds in Table 1.

The mass spectra in negative ion mode of the 80% ethanolic extract are shown in Figure 2. Related mass data (e.g., *m*/*z*, molecular formula, ion fragments) are presented in Table 1 for the identification of these substances. When comparing the mass data of the ethanolic extract to the mass data obtained from the literature, phytochemical standards or mass bank, 14 substances were identified, two of which were organic acids and 12 were phenols. Among those 12 phenolic compounds, five substances belonged to gallic acid and its derivatives (compounds **3**–**7**) and the remaining seven compounds were flavonoids and its derivatives (compounds **8**, **9**, **10**, **11**, **12**, **13** and **14**). Table 1 summarizes the quantitative and semi-quantitative results of 14 phytochemical compounds. The malic acid (144,519.79 ± 21,651.25 µg/g) and citric acid (135,452.78 ± 16,530.37 µg/g) comprised the most content, both of which are organic acids. The phenolic compound with the highest content was gallic acid (3791.02 ± 490.83 µg/g), followed by quercetin-3-*O*-rhamnoside (quercitrin, 3592.77 ± 463.06 µg/g) and myricetin-3-*O*-rhamnoside (525.43 ± 64.31 µg/g), indicating that those three substances were the principal phenolic compounds of the ethanolic extract of *R. chinensis* fruits.

### 3.2. Network Pharmacology Analysis

**Figure 3:**
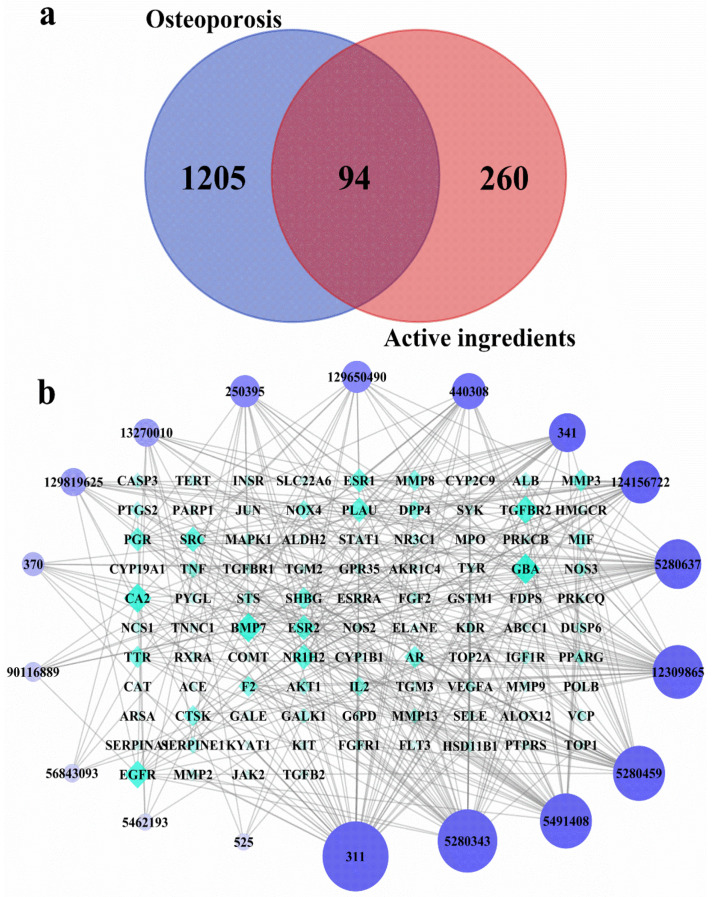
Screening of intersecting targets and construction of network diagrams of chemical components and targets. (**a**) A Venn diagram showing the intersections of identified targets of identified compounds and osteoporosis; (**b**) The identified compounds–osteoporosis target network. The color and size of each icon reflects the node degree for the common targets. The active ingredients include: Malic acid (525, Peak 1), Citric acid (311, Peak 2), Gallic acid (370, Peak 3), Digallic acid (341, Peak 4), 1,2,3-tri-*O*-galloyl-d-glucose (13270010, Peak 5 or 6 or 7), 1,2,6-trigalloyl-beta-d-glucose (124156722, Peak 5 or 6 or 7), 1,2,6-Trigalloyl-glucose (440308, Peak 5 or 6 or 7), 1,3,6-tri-*O*-galloylglucose (250395, Peak 5 or 6 or 7), 1,4,6-Trigalloylglucose (129650490, Peak 5 or 6 or 7), 2,3,6-trigalloyl-d-glucose (129819625, Peak 5 or 6 or 7), Trigalloylglucose (90116889, Peak 5 or 6 or 7), Myricetin-3-*O*-galactoside (5491408, Peak 8), Myricetin-3-*O*-rhamnoside (56843093, Peak 9), Luteolin-7-*O*-glucoside (5280637, Peak 10), Quercetin-3-*O*-arabinoside (12309865, Peak 11), Quercetin-3-*O*-rhamnoside (5280459, Peak 12), Kaempferol-3-*O*-hexoside (5462193, Peak 13), Quercetin (5280343, Peak 14).

A total of 354 potential targets of the identified substances in the ethanolic extract *R. chinensis* fruits were predicted by using TCMSP, Swiss Target Prediction, and PharmMapper databases. After searching from DrugBank, GeneCards, and TTD databases, 1299 targets related to OP were identified. By comparing potential targets of the identified substances with OP-related targets, 94 intersection targets were determined as the possible targets of the ethanolic extract towards OP (Figure 3a). A compound–target network was established to identify the correlation between the identified compounds and their potential targets (Figure 3b). Citric acid, quercetin, myricetin-3-*O*-galactoside and quercetin-3-*O*-rhamnoside were the four compounds most associated with OP-related targets (**44**, **39**, **33** and **33** OP-related targets, respectively). Meanwhile, citric acid and quercetin-3-*O*-rhamnoside were the predominant phytochemicals in the extract (Figure 2 and Table 1). However, malic acid and gallic acid were also the most abundant compounds contained in the extract, but these two substances were associated with fewer OP-related targets (5 and 10 OP-related targets, respectively).

**Figure 4:**
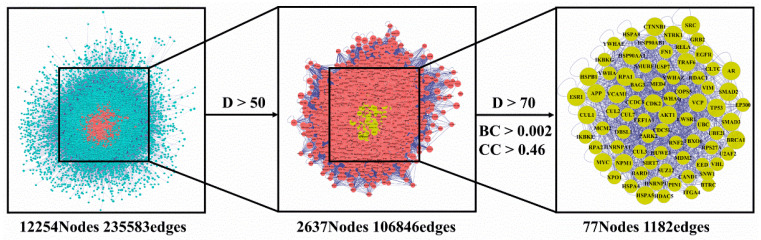
Common targets of compounds and osteoporosis PPI network. PPI, protein–protein interaction; D, Degree; BC, Betweenness Centrality; CC, Closeness Centrality.

**Figure 5:**
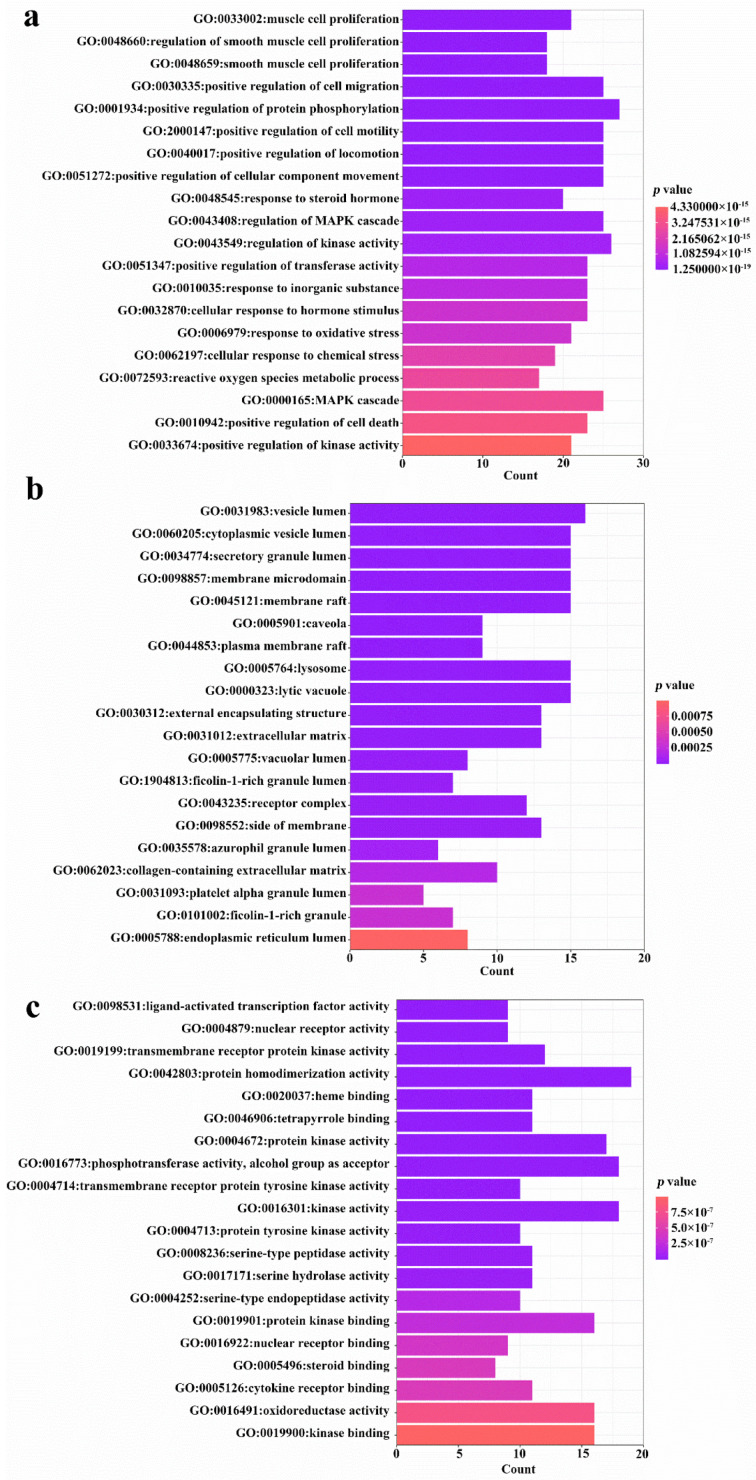
GO enrichment analysis of 94 common targets (*p*-value < 0.05). (**a**) Top 20 significantly enriched GO terms in “biological process” (BP); (**b**) “cellular component” (CC); and (**c**) “molecular function” (MF) are shown. Enrichment scores represent-log *p* values.

**Figure 6:**
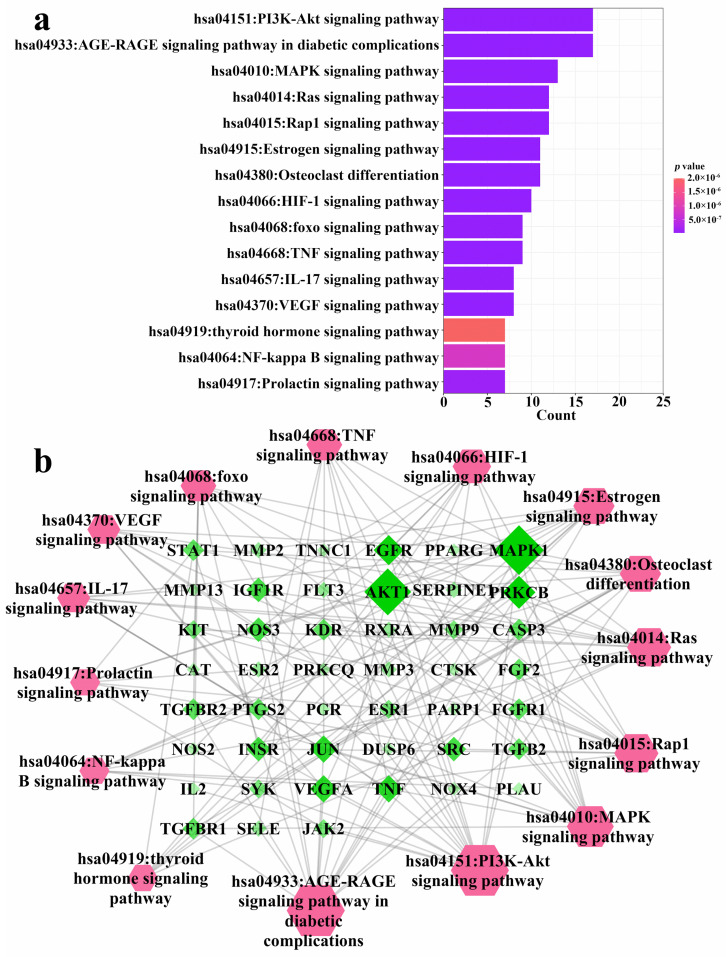
KEGG pathway enrichment analysis of 94 common targets (*p*-value < 0.05) and the gene–pathway network of the identified compounds against osteoporosis. (**a**) KEGG pathways; (**b**) Gene–pathway network. The color and size of each icon reflects the node degree for the common targets.

The 94 intersection targets were further analyzed by Bisogenet to construct a PPI network, and the results are shown in Figure 4. Altogether 77 important nodes were identified in the PPI network. The top 20 enriched GO terms in each category are shown in Figure 5. The screened targets mainly involved biological processes, including “proliferation of muscle cell (GO:0033002)”, “regulation of proliferation of smooth muscle cell (GO:0048660)” and “proliferation of smooth muscle cell (GO:0048659)”. While “vesicle lumen (GO:0031983)”, “cytoplasmic vesicle lumen (GO:0060205)” and “secretory granule lumen (GO:0034774)” ranked the highest in the molecular function category, “activity of ligand-activated transcription factor (GO:0098531)”, “nuclear receptor activity (GO:0004879)”, and “activity of transmembrane receptor protein kinase (GO:0019199)” were the main cellular component categories. The crucial signaling pathways involved were further illuminated by enrichment analysis of KEGG pathway (Figure 6). The top three KEGG pathways (Figure 6a) were the PI3K-Akt (hsa04151), AGE-RAGE (hsa04933), and MAPKs (hsa04010) signaling pathways. In accordance with the number of targets found in each signaling pathway, Cytoscape (3.8.2) software was applied to establish a target–signal pathway network. The target–pathway network consisted of 60 nodes and 158 edges (Figure 6b). The results showed that Mitogen-activated protein kinase 1 (MAPK1) and RAC-alpha serine/threonine-protein kinase (AKT1) were the two most critical targets, which involved 14 and 13 pathways, respectively. Combined with PPI and enrichment analysis of KEGG pathways and the target–signaling pathway network, AKT1 and MAPK1 are relatively key targets. They are associated with more pathways (Figure 6b) and interact closely with other targets (Figure 4). Among all phytochemical components, quercetin-3-*O*-rhamnoside and quercetin-3-*O*-arabinoside inhibited both targets. Citric acid, quercetin, and 1,2,6-trigalloyl-beta-d-glucose inhibited AKT1, while myricetin-3-*O*-galactoside inhibited MAPK1.

### 3.3. Inhibition of Osteoclast Differentiation

**Figure 7:**
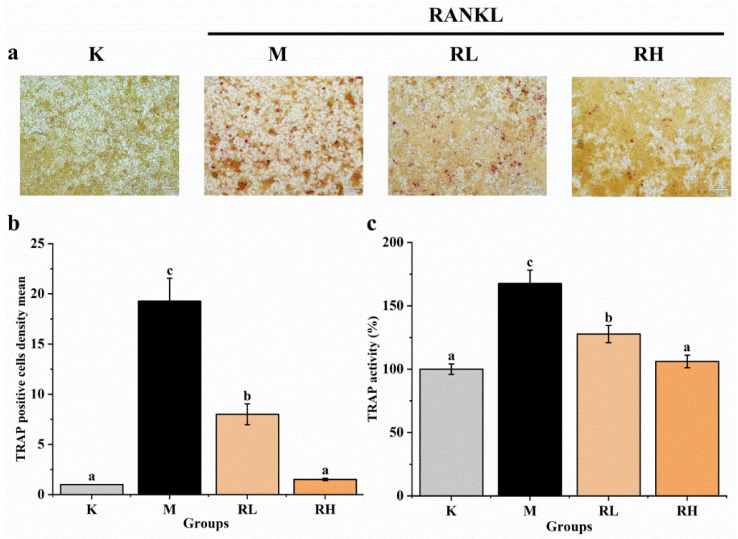
Effects of the ethanolic extract from *R. chinensis* fruits on RANKL-induced osteoclastogenesis in RAW264.7 cells. (**a**) TRAP staining; (**b**) Relative area of TRAP-positive signal, normalized with the K group; (**c**) Quantitative results of TRAP activity. All the values are expressed as mean ± SD (*n* = 3). Groups with different letters are significantly different (*p* < 0.05). The TRAP positive cells after TRAP staining were quantified using Image-Pro Plus software. The quantitative result is expressed in terms of density mean (density mean = density sum/area sum) and the TRAP positive cells were quantified by normalizing with group K. K, the Control group; M, 50 ng/mL RANKL; RL, 50 ng/mL RANKL and 50 µg/mL of ethanolic extract; RH, 50 ng/mL RANKL and 100 µg/mL of ethanolic extract; TRAP, tartrate-resistant acid phosphatase; RANKL, receptor activator of nuclear factor-κB ligand.

After five days of RANKL induction, the cells were incubated with or without ethanolic extract and stained with a TRAP staining kit, and the relative area of TRAP-positive cells was quantified with image pro plus software. At the same time, a TRAP activity kit was used to determine the TRAP activity of cells. As shown in Figure 7a, RAW264.7 cells in the M group differentiated into a large number of TRAP-positive osteoclasts. After adding 50 μg/mL (the RL group) and 100 μg/mL (the RH group) ethanolic extracts, the formation of TRAP-positive osteoclasts was clearly inhibited. As shown in Figure 7b, when compared to the M group, the TRAP-positive osteoclasts in the RL and RH groups had evidently declined (*p* < 0.05), remarkably in the RH group, in which the TRAP-positive osteoclasts were similar to those in the K group (*p* > 0.05). It can be seen from the results of the TRAP activity (Figure 7c) that the TRAP activity of the M group increased when compared with that of the K group (*p* < 0.05), while the TRAP activities of the RL and RH groups were remarkably lower than the M group (*p* < 0.05), and no difference in TRAP activity between the RH and K groups was observed (*p* > 0.05), which were consistent with the staining results.

### 3.4. Ethanolic Extract Inhibits Osteoclastogenesis through MAPKs, NF-κB and Akt Signaling Pathways

**Figure 8:**
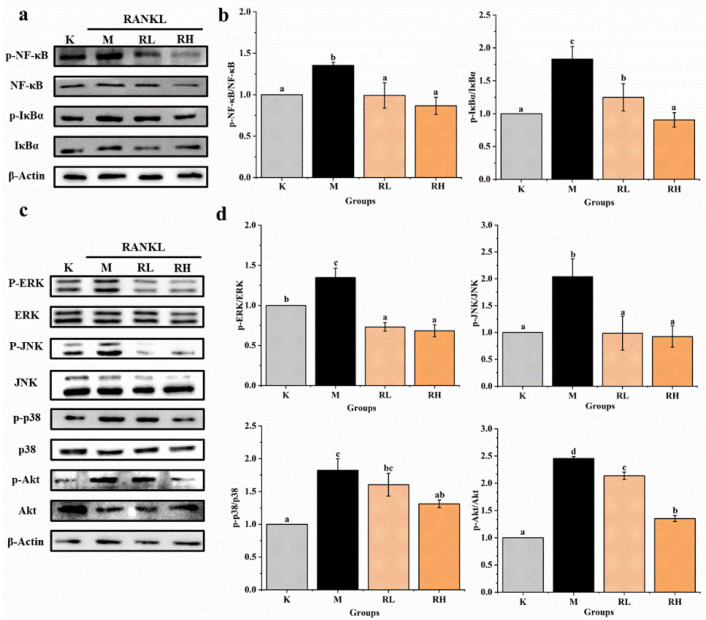
Effects of the ethanolic extract from R. chinensis fruits on RANKL-induced osteoclastogenesis to NF-κB, Akt, and MAPKs signaling pathways in RAW264.7 cells. (**a**) Western blot analysis of p-NF-κB/NF-κB and p-IκBα/IκBα proteins; (**b**) the relative expression of p-NF-κB/NF-κB and p-IκBα/IκBα proteins was quantified by normalization with group K and β-actin according to grayscale; (**c**) Western blot analysis of p-ERK/ERK, p-JNK/JNK, p-p38/p38 and p-Akt/ Akt proteins; (**d**) the relative expression of p-ERK/ERK, p-JNK/JNK, p-p38/p38 and p-Akt/Akt proteins was quantified by normalization with group K and β-actin according to grayscale. All the values are expressed as mean ± SD (*n* = 3). Groups with different letters mean significantly different (*p* < 0.05). K, the Control group; M, 50 ng/mL RANKL; RL, 50 ng/mL RANKL and 50 µg/mL of ethanolic extract; RH, 50 ng/mL RANKL and 100 µg/mL of ethanolic extract; RANKL, receptor activator of nuclear factor-κB ligand; p-NF-κB/NF-κB, phosphorylated-nuclear factor κB/nuclear factor κB; p-IκBα/ IκBα, phosphorylated-inhibitor α of nuclear factor κB/ inhibitor α of nuclear factor κB; p-ERK/ERK, phosphorylated-extracellular regulated protein kinases/extracellular regulated protein kinases; p-JNK/JNK, phosphorylated-Jun N-terminal kinase /Jun N-terminal kinase; p-P38/P38, phosphorylated-P38 mitogen-activated protein kinase/P38 mitogen-activated protein kinase; p-Akt/Akt, phosphorylated-protein kinase B/protein kinase B.

Western blot assays of several signaling pathways were used to further confirm the possible mechanisms of *R. chinensis* fruits on RANKL-induced osteoclastogenesis, and the results are presented in Figure 8. After induction by RANKL for 5 days, the levels of some key proteins (p-NF-κB, NF-κB, p-IκBα, IκBα, p-ERK, ERK, p-JNK, JNK p-p38, p38, p-Akt, Akt) in cells were determined. The expression of p-NF-κB/NF-κB, p-IκBα/IκBα, p-ERK/ERK, p-JNK/JNK, p-p38/p38 and p-Akt/Akt in the M group were significantly higher than their counterparts in the K group (*p* < 0.05). The expression of almost all proteins in the RL group and RH group were obviously less than that of the relevant protein in the M group (*p* < 0.05), except for p-p38/p38 in the RL group (Figure 8b,d).

### 3.5. Inhibition of c-Fos and NFATc1 Expression by Ethanolic Extract

**Figure 9:**
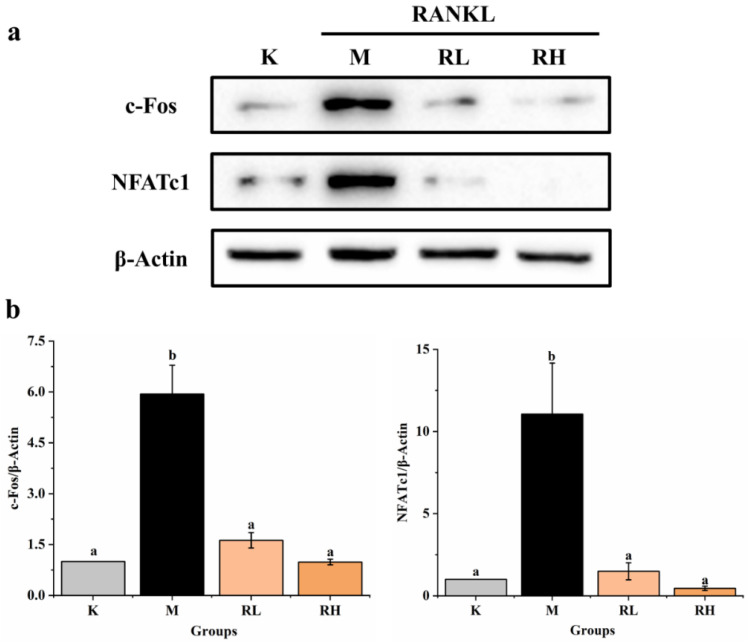
Effects of the ethanolic extract from the *R. chinensis* fruits on RANKL-induced osteoclastogenesis on c-Fos and NFATc1 proteins in RAW264.7 cells. (**a**) Western blot analysis of c-Fos and NFATc1 proteins; (**b**) the relative expression of c-Fos and NFATc1 proteins was quantified by normalization with group K and β-actin according to grayscale. All the values are expressed as mean ± SD (*n* = 3). Groups with different letters are significantly different (*p* < 0.05). K, the Control group; M, 50 ng/mL RANKL; RL, 50 ng/mL RANKL and 50 µg/mL of ethanolic extract; RH, 50 ng/mL RANKL and 100 µg/mL of ethanolic extract; RANKL, receptor activator of nuclear factor-κB ligand; NFATc1, nuclear factor of activated T-cells cytoplasmic 1.

As shown in Figure 9, the expressions of both c-Fos and NFATc1 in group M were significantly higher than that of the corresponding protein in Group K (*p* < 0.05). However, the expression levels of c-Fos and NFATc1 (Figure 9b,c) in the RL group and RH group were significantly less when compared to the corresponding protein in the M group (*p* < 0.05). According to these results, we can calculate that the ethanolic extract of the fruits could efficiently suppress NFATc1 and c-Fos protein expression during the RANKL induction period, indicating that NFATc1 and c-Fos may also be one of its potential targets for inhibiting osteoclastogenesis.

## 4. Discussion

In this study, 14 components were detected in the ethanolic extract of *R. chinensis* fruits, including 2 kinds of organic acids and 12 kinds of polyphenols. Network pharmacology analysis showed that citric acid, quercetin, myricetin-3-*O*-galactoside, and quercetin-3-*O*-rhamnoside may be the main potential bioactive components of *R. chinensis* fruits that inhibit osteoclastogenesis, and the main potential targets are AKT1 and MAPK1, and the main potential pathways are PI3K-Akt, AGE-RAGE and MAPKs signaling pathways. Cell experiments and western blotting further verified that the ethanolic extract of the fruits could effectively inhibit the differentiation and formation of osteoclasts, which maybe by regulating the NF-κB, Akt, and MAPKs signaling pathways, as well as downregulating the expression levels of c-Fos and NFATc1 proteins.

Overexpression of osteoclasts can cause progressive bone loss, which in turn leads to osteoporosis, and may even lead to bone fragility and fractures, which endanger human health [35]. Therefore, it is very important to explore effective natural products to inhibit osteoclastogenesis. Many studies have shown that polyphenols may have an advantageous effect on bone metabolism [36,37]. Foods rich in vegetables, fruits and whole grains were correlated with a lower danger of falls and fractures and an increase in bone density [38]. However, the differentiation of osteoclasts is influenced by multiple signaling pathways, in a complex process [39]. In order to further screen the anti-osteoporosis active components in *R. chinensis* fruits, as well as the main action pathways and related targets, the method of network pharmacology was used. Network pharmacology can predict active ingredients that are highly related to osteoporosis targets. At the same time, network pharmacology can also predict the potential main targets and pathways of these active ingredients. Generally, the network pharmacology results showed that *R. chinensis* fruits may inhibit osteoclastogenesis by inhibiting AKT1 and MAPK1 involved in the PI3K-Akt and MAPKs signaling pathways, respectively (Figure 4 and Figure 6). Citric acid, quercetin, myricetin-3-*O*-galactoside, and quercetin-3-*O*-rhamnoside were thought to be the dominant active ingredients for the anti-OP effect of *R. chinensis* fruits (Figure 3). Choi et al. [40], reported that quercetin-3-*O*-rhamnoside reversed oxidative stress-induced osteoblast dysfunction. The concentration of citric acid in bone is roughly 50-fold higher than that in most soft tissues, suggesting that citric acid plays a major role in the textural properties or features of bone, and serum citric acid level may be a marker of bone loss related diseases; serum citric acid level in OP in animals was significantly lower [13]. In addition, Kim et al. [23], reported that quercetin inhibited osteoclastogenesis, reduced RANKL levels, and decreased RANKL and IL-17-induced differentiation of monocytes to osteoclasts in multiple ways, and could be potentially used as an alternative therapeutic agent in regulating bone destruction and inflammation in rheumatoid arthritis. In addition, those pathways, such as PI3K-Akt, AGE-RAGE, MAPKs, NF-κB and thyroid hormone signaling pathways have been confirmed to be associated with the prevention and/or treatment of OP [4,41,42]. Protein NF-κB (NF-κB) is known to be associated with several metastatic bone diseases [43], and osteoclast formation [44]. Osteoclast differentiation requires RANK ligands to bind to receptors and subsequently activate multiple intracellular pathways, including AKT/PI3K, MAPK, and NF-κB, which in turn leads to osteoclast formation [45]. Besides the NF-κB and MAPKs signaling pathways, activation of the PI3K/Akt pathway also plays a key role in the formation of osteoclasts [46]. The PI3K/Akt signaling pathway activated by RANKL has been proved to play a crucial role in regulating osteoclast survival and differentiation [47]. According to the findings of network pharmacological analysis and previous literature, the effect of *R. chinensis* fruits on suppressing RANKL-induced osteoclastogenesis may involve PI3K-Akt, MAPK, and NF-κB signaling pathways.

TRAP is an iron-binding protein that can induce osteoclast differentiation, and high expression levels of TRAP are commonly observed during osteoclast differentiation [48]. It was also found that TRAP could affect the functional activity of osteoclasts by modulating bone matrix absorption and collagen conversion [49]. TRAP is reported to be involved in the migration of osteoclasts to bone adsorption sites, which is the main cause of OP [50]. Therefore, the highly expressed TRAP in osteoclasts is generally used as a phenotypic marker of osteoclasts [51]. Therefore, the ethanolic extract of *R. chinensis* fruits could effectively suppress the differentiation of osteoclasts (Figure 7). Previously, polyphenols have also been reported to reduce TRAP activity in RANKL-induced RAW264.7 cells. For example, Suh et al. [52], showed that xanthohumol significantly inhibited TRAP activity in RANKL-stimulated RAW264.7 cells, and Lee et al. [53], reported that the extract of *Ramalina litoralis,* rich in phenolic compounds, significantly reduced the mRNA expression of TRAP.

As shown in Figure 8, the ethanolic extract of the fruits inhibited RANKL-stimulated osteoclast formation by inhibiting MAPKs, NF-κB, and Akt signaling pathways, which is consistent with the above prediction results of network pharmacology analysis. MAPKs and NF-κB signaling pathways play a crucial role in the differentiation and formation of osteoclasts, and the MAPKs cascade is activated by phosphorylating p38, ERK1/2, and JNK [54,55]. Those signals interact with NFATc1, the main regulator of osteoclast differentiation, to induce nuclear transfer, and thereby promote osteoclastogenesis [56]. Many previous studies have also reported that many natural products inhibit osteoclastogenesis and prevent OP by inhibiting MAPKs and NF-κB signaling pathways. For example, in the study of Hou et al. [57], the expressed levels of NF-κB was significantly reduced in OP rats after being treated with ferulic acid, and ferulic acid showed a good ability to prevent OP in neonatal rats. Choi et al. [5] reported that Platycodin D inhibited osteoclast differentiation by inhibiting NF-κB and ERK/p38 MAPK signaling pathways. Ang et al. [58], also found that naringin could suppress NF-κB activation and ERK phosphorylation in RANKL-stimulated cells.

In addition, protein c-Fos is intimately related to the occurrence and growth of osteosarcoma, and in the process of osteoclast division, c-Fos is an important adjusting factor of RANKL downstream, which promotes osteoclast shaping mainly by activating the downstream factor NFATc1 [59]. Madhuri et al. [60], also reported that 100 μM of ferulic acid markedly inhibited the expressions of c-Fos and NFATc1 genes induced by RANKL and M-CSF. In addition, it is reported that Tatarinan O, a substance obtained from the *Acorus tatarinowii Schott* roots, could effectively attenuate osteoclastogenesis from RANKL-induced BMMs via lowering the expressed levels of c-Fos and NFATc1 [61]. *R. chinensis* fruits could effectively inhibit the expression of NFATc1 and c-Fos proteins during the RANKL-induced period. This indicated that the *R. chinensis* fruits have a good inhibitory activity on the differentiation and formation of osteoclasts. In the future, its function in healthcare of diseases related to osteoclast differentiation can be studied to explore its further utilization.

## 5. Conclusions

Results of the current work showed that *R. chinensis* fruits have a preventive effect on the formation of osteoclasts in RAW264.7 cells induced by RANKL, which may involve multiple targets and multiple pathways. Among the 14 identified compounds, citric acid, quercetin, myricetin-3-*O*-galactoside, and quercetin-3-*O*-rhamnoside may be the main active components that inhibit osteoclastogenesis. *R. chinensis* fruits significantly inhibited osteoclast formation via adjusting the NF-κB, Akt, and MAPKs signaling pathways, and by downregulating the expressed levels of c-Fos and NFATc1 proteins. In addition, the main potential targets of *R. chinensis* fruits that inhibit osteoclastogenesis are AKT1 and MAPK1, and the main potential signaling pathways may be AGE-RAGE and PI3K-Akt signaling pathways. This study may supply a basis for further use of *R. chinensis* fruits as a functional food and/or an alternative method for the prevention and improvement of osteoporosis and related diseases.

## Figures and Tables

**Table 1:** Chemical composition identified in *R. chinensis* Mill. fruits by UHPLC-ESI-HRMS/MS.

Peak No.	RT (Min)	Compounds	[M − H]^−^ (*m*/*z*)	Molecular Formula	MS/MS Fragment Ions	Dry Extract (µg/g)	Reference
1	1.37	Malic acid	133.0130	C_4_H_6_O_5_	115.1210(100)	144,519.80 ± 21,651.25	Standard
2	1.80	Citric acid	191.0189	C_6_H_8_O_7_	87.0075(100), 57.0332(91), 111.0075(48)	135,452.78 ± 16,530.37	Standard
3	2.58	Gallic acid	169.0133	C_7_H_6_O_5_	69.0331(100), 124.0152(55), 125.0232(33)	3791.02 ± 490.83	Standard
4	9.55	Digallic acid	321.0252	C_14_H_10_O_9_	125.0232(100), 169.0133(23)	148.89 ± 20.36	[33]
5	9.81	Trigalloyl glucose isomer I	635.0894	C_27_H_24_O_18_	169.0134(100), 483.0779(16), 635.0867(2)	108.30 ± 13.08	[34]
6	10.17	Trigalloyl glucose isomer II	635.0895	C_27_H_24_O_18_	169.0133(100), 483.0778(9), 635.0895(4)	167.08 ± 22.72	[34]
7	11.30	Trigalloyl glucose isomer III	635.0893	C_27_H_24_O_18_	169.0134(100), 483.0783(15), 635.0930(4)	326.75 ± 40.48	[34]
8	14.11	Myricetin-3-*O*-galactoside	479.0838	C_21_H_20_O_13_	316.0224(100), 317.0271(26)	61.27 ± 6.97	Mass bank
9	16.10	Myricetin-3-*O*-rhamnoside	463.0886	C_21_H_20_O_12_	316.0225(100), 317.0276(24)	525.43 ± 64.31	Standard
10	17.07	Luteolin-7-*O*-glucoside	447.0933	C_21_H_20_O_11_	285.0402(100), 284.0327(53)	81.57 ± 10.60	Standard
11	17.90	Quercetin-3-*O*-arabinoside	433.0775	C_20_H_18_O_11_	300.0275(100), 301.0331(17)	50.69 ± 7.59	[33]
12	18.92	Quercetin-3-*O*-rhamnoside	447.0931	C_21_H_20_O_11_	300.0276(100), 301.0341(54), 151.0029(23)	3592.77 ± 463.06	Standard
13	21.37	Kaempferol-3-*O*-hexoside	431.0984	C_21_H_22_O_10_	284.0326(96), 285.0397(68)	177.93 ± 21.13	[34]
14	24.91	Quercetin	301.0354	C_15_H_10_O_7_	151.0026(100)	173.38 ± 24.54	Standard

RT: retention time; the results were expressed as µg/g of dry extract. All the values are expressed as mean ± SD (*n* = 3). Malic acid standard was used for quantifying compounds 1; Citric acid standard was used for quantifying compounds 2; gallic acid standard was used for quantifying compounds **3**, **4**, **5**, **6**, **7**; myricetin-3-*O*-rhamnoside standard was used for quantifying compounds **8**, **9**; luteolin-7-*O*-glucoside standard was used for quantifying compound **10**; quercetin-3-*O*-rhamnoside standard was used for quantifying compounds **11** and **12**; kaempferol standard was used for quantifying compound **13**; quercetin standard was used for quantifying compound **14**.

## Data Availability

The data that support the findings of this study are available from the corresponding author upon reasonable request.
